# Exploring Vascular Complications in Ovarian Cancer Surgery: A Narrative Literature Review with a New Management Proposal Algorithm

**DOI:** 10.3390/healthcare13030270

**Published:** 2025-01-30

**Authors:** Matilde Degano, Martina Arcieri, Paolo Frigatti, Paola Scrivere, Silvia Zermano, Lorenza Driul, Giuseppe Cucinella, Carlo Ronsini, Marco Petrillo, Giampiero Capobianco, Guglielmo Stabile, Chiara Ripepi, Stefano Cianci, Stefano Uccella, Vito Chiantera, Giovanni Scambia, Giuseppe Vizzielli, Stefano Restaino

**Affiliations:** 1 Clinic of Obstetrics and Gynecology, Santa Maria della Misericordia University Hospital, Azienda Sanitaria Universitaria Friuli Centrale, 33100 Udine, Italy; matilde.degano@asufc.sanita.fvg.it (M.D.); martina.arcieri@asufc.sanita.fvg.it (M.A.); silvia.zermano@asufc.sanita.fvg.it (S.Z.);; 2 Unit of Vascular and Endovascular Surgery, General Surgery Department, Santa Maria della Misericordia University Hospital, Azienda Sanitaria Universitaria Friuli Centrale, 33100 Udine, Italy; paolo.frigatti@asuiud.sanita.fvg.it (P.F.); paola.scrivere@asufc.sanita.fvg.it (P.S.); 3 Department of Medicine, University of Udine, 33100 Udine, Italy; 4 Gynecologic Oncology Unit, Istituto Nazionale Tumori IRCCS Fondazione G. Pascale, 80131 Naples, Italy; 5 Department of Woman, Child and General and Specialized Surgery, University of Campania Luigi Vanvitelli, 80138 Naples, Italy; 6 Gynecologic and Obstetric Clinic, Department of Medicine, Surgery and Pharmacy, University of Sassari, 07100 Sassari, Italy; mpetrillo@uniss.it (M.P.); capobia@uniss.it (G.C.); 7 Institute for Maternal and Child Health IRCCS Burlo Garofolo, 34137 Trieste, Italy; guglielmo.stabile@burlo.trieste.it (G.S.); chiara.ripepi@burlo.trieste.it (C.R.); 8 Unit of Gynecology and Obstetrics, Department of Human Pathology of Adult and Childhood G. Barresi, University of Messina, 98122 Messina, Italy; stefano.cianci@unime.it; 9 Department of Obstetrics and Gynecology, Azienda Ospedaliera Universitaria Integrata Verona, 37126 Verona, Italy; stefano.uccella@univr.it; 10 Unit of Gynecologic Oncology, ARNAS Civico—Di Cristina—Benfratelli, Department of Health Promotion, Mother and Child Care, Internal Medicine and Medical Specialties (PROMISE), University of Palermo, 90127 Palermo, Italy; vito.chiantera@unipa.it; 11 Department of Women's and Children's Health Sciences and Public Health, Fondazione Policlinico Universitario A. Gemelli IRCCS, 00169 Rome, Italy; 12 PhD School in Biomedical Sciences, Gender Medicine, Child and Women Health, University of Sassari, 07100 Sassari, Italy

**Keywords:** ovarian cancer surgery, vascular surgery, vascular complications

## Abstract

Background/Objectives: Vascular complications during ovarian cancer surgery are rare but potentially severe. The objective of this review was to underline the need to standardize and optimize the management of these rare complications within an evidence-based framework. Methods: This review included the literature until 29 December 2024 and finally analyzed 17 studies, with 40 vascular complications reported. Results: Complications primarily occurred intraoperatively and involved both arterial and venous systems. Management approaches involved interdisciplinary collaboration, including vascular surgeons and interventional radiologists. Nevertheless, the collected data do not accurately reflect the reality of vascular complications in ovarian cancer surgery, as more than half of the included studies were case reports or research letters. This highlights the lack of standardized guidelines and limited training in vascular surgery for gynecologic oncologists, the importance of preoperative planning, including detailed imaging, risk stratification, and a multidisciplinary approach to mitigate complications. The authors propose an algorithm emphasizing prevention, timely identification, and effective management of vascular injuries alongside postoperative monitoring. Conclusions: The findings stress the need for treatment in high-volume tertiary centers and advocate advanced surgical training, incorporating virtual reality simulations to address vascular complications. Future research should focus on large multicenter studies to establish evidence-based guidelines for managing vascular complications in ovarian cancer surgery. Innovations in technology and education may further improve outcomes, ensuring optimal care for patients undergoing these complex procedures.

## 1. Introduction

Vascular damages in gynecologic cancer surgery are a rare condition with potentially fatal complications. The reported incidence in various studies is less than 1% [1,2,3,4,5,6,7]. The overall mortality from vascular iatrogenic injury is reported to be up to 18% [8]. Vascular repairs during gynecologic surgery, both for oncologic and benign pathologies, independently predict increased morbidity and mortality [9].

Due to their anatomical variability and deep location, the iliac veins are the most injured vessels in gynecologic surgery [10]. Other vessels frequently at risk of iatrogenic injury in gynecologic oncologic surgery include the aorta, inferior vena cava, iliac arteries, gonadal vessels, and mesenteric vessels [2].

Vascular injuries can occur during both laparotomic and mini-invasive surgery. In laparoscopy, the initial entry constitutes a pivotal moment irrespective of the technique employed. However, adverse effects may also occur during trocar placement, sharp dissection, or other related electrosurgery procedures [1,5,11,12,13]. In addition, surgery for gynecologic cancers often involves radicalization procedures that encompass non-gynecologic pelvic and abdominal districts. This is particularly true for ovarian carcinoma, given the extent of the disease. In cytoreductive surgery for ovarian cancer, for example, the complications will naturally be those associated with the upper abdominal surgery performed in the specific case [14,15], foremost among them the vascular district surgery. The primary vessel’s infiltration increases the risk of vascular damage but is not considered a contraindication for radical surgery. However, very accurate and multidisciplinary preoperative planning is even more necessary in these cases. Complete eradication of the tumor may necessitate removing vascular structures with or without subsequent reconstruction, or it may involve the lymph node district, which is intrinsically and anatomically close to the vascular one [16]. The surgical procedure most frequently cited in cases of vascular complications in gynecologic oncology surgery is indeed lymphadenectomy, a fundamental step in the surgical approach to all major gynecologic tumors [10,17,18].

This close correlation between gynecologic oncologic surgery and vascular surgery has recently been highlighted by the emergence of a new term to define this approach: “oncovascular surgery”. It requires adequate surgical planning and a multidisciplinary approach, often involving vascular surgeons [19,20].

The precise incidence of complications specifically associated with ovarian cancer surgery is significantly less defined in the literature compared to vascular complications in gynecological surgery overall or laparoscopy in general. This also applies to the types of vessels most frequently affected and other relevant information regarding management strategies. Indeed, due to the rarity of this occurrence, most studies that address this subject tend to analyze gynecologic cancers as a whole. The objective of this review arises specifically from the limited information available concerning vascular complications unique to ovarian cancer surgery and the need to standardize and optimize the management of these rare complications within an evidence-based framework. The aim was, therefore, to investigate the information available in the literature regarding vascular complications in ovarian cancer surgery, specifically focusing on the type of injury, the vessels involved, the presence or absence of anatomical variants, the type of surgical approach during which the complication occurred, the management, and the outcome. The second aim was to design, based on the data available or lacking in the literature and expert opinions, a management algorithm for these complications that could assist gynecologic surgeons in improving patient outcomes.

## 2. Materials and Methods

We conducted this review according to the Preferred Reporting Items for Systematic Reviews and Meta-Analyses (PRISMA) guidelines. We searched for studies via PubMed and Scopus, using the following search terms. In SCOPUS we used the following: ovarian AND cancer AND vascular AND injury, ovarian AND cancer AND vascular AND surgical AND complications, ovarian AND cancer AND vascular AND intraoperative AND complications, ovarian AND cancer AND vascular AND postoperative AND complications; in PUBMED we used the following: (ovarian cancer) AND (vascular injury), (ovarian cancer) AND (vascular surgical complications), (ovarian cancer) AND (vascular intraoperative complications), (ovarian cancer) AND (vascular postoperative complications). The search was extended until 29 December 2024. This strategy resulted in many doubles, which were deleted at the beginning of the process. Titles and abstracts were independently screened for inclusion by 2 investigators (M.D. and S.Z.). Full-text versions of eligible articles were reviewed by 2 investigators (M.D. and S.Z.) to determine eligibility; any questions regarding the inclusion of studies or discordance in screening were then resolved by senior authors (G.V., S.R., and M.A.). All articles (case reports, case series, clinical trials, and reviews) describing vascular complications in ovarian cancer surgery were included. Articles written in non-English languages were excluded, such as those other than case reports/case series, with outcomes of interest not reported or out of topic. The authors believe it is important to emphasize that many studies were excluded because they dealt with vascular complications in gynecological cancers as a whole, and from the data presented, it was not possible to extract specific information on ovarian carcinoma alone. Among the included articles, some still address all gynecological cancers, but some data were extractable for ovarian carcinoma patients only. The authors of that work were contacted via e-mail to inquire about access to the remaining data, but no response was received. Studies concerning managing vascular neoplastic invasion without complications were excluded, as this is not a complication but rather an independent risk factor for vascular complications themselves. One of the included articles [21] dates to 1975. Given the scarcity of included articles with the chosen criteria, the authors decided not to use the temporal criterion for exclusion.

Finally, we analyzed a subset of 17 articles. Data extraction was performed by one of the same investigators who screened the articles (M.D.). The data were confirmed twice by the second reviewer (S.Z.) to minimize potential errors. Conflicts were resolved by consensus and discussion with senior authors (G.V., S.R., and M.A.). We extracted information on number of patients included in the study, number of vascular complications that occurred, whether the complication was intra- or postoperative, if the pathology was primary or recurrence, the postoperative complication grade (based on Clavien–Dindo classification [22]), type of surgery (e.g., cytoreductive vs. diagnostic), surgical procedure (e.g., lymphadenectomy), type of vessel involved, need for transfusions, management, involvement of vascular surgeon or other specialists, vascular anomalies founded intra or preoperative, patient age, comorbidities, previous surgery, robotic/laparoscopic/laparotomic approach, need for laparotomic conversion, type of vessel lesion, tumor laterality, stage and histotype, blood loss, outcome.

## 3. Results

**Figure 1:**
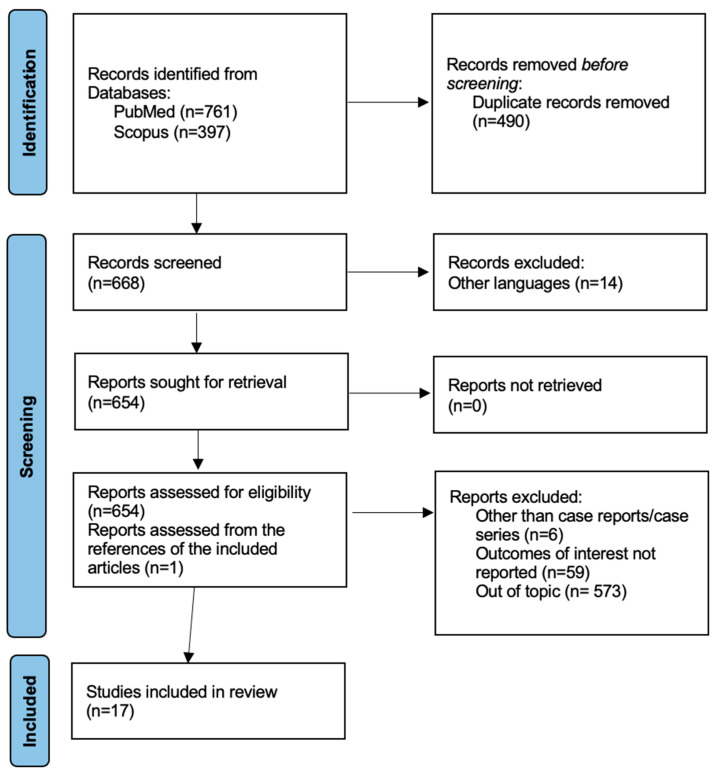
PRISMA 2020 flowchart.

The initial search identified 1158 studies (761 on PubMed and 397 on Scopus). First, 490 duplicates were removed, resulting in 668 articles to screen. Then, 14 articles were excluded because they were written in a non-English language. Another 638 articles were then excluded because other than case report/case series (n = 6), the outcome of interest was not reported (n = 59) or because it was out of topic (n = 573) (Figure 1). Additionally, an article that had been missed in the PubMed and Scopus searches was found and deemed suitable for inclusion through the references of the included articles. The remaining 17 articles were then analyzed.

The collected studies include nine case reports, two research letters, five monocentric retrospective studies, and one multicentric retrospective study, dating from 1975 to 2024 (Table 1). In total, among these studies, 1456 patients undergoing surgery for ovarian cancer were reported, with vascular complications noted in 40 cases. These data do not accurately reflect the true incidence of vascular complications in ovarian cancer surgery, as more than half of the included studies report isolated cases of vascular complications as case reports or research letters. The median age of the patients is 62 years, but this is not precise data since, in some of the studies considered, the reported median age was that of the entire group of patients and not just of those who experienced vascular complications. Additionally, in one study, the age was not recorded. Data on comorbidities and Body Mass Index (BMI) of complication patients were obtained in only six studies. One patient was recorded with type II diabetes and a BMI of 31.6 kg/m^2^; another was obese with a BMI of 30 kg/m^2^; one had well-controlled hypertension; one had hypertension and osteoporosis; and two patients had no associated comorbidities. In the remaining cases, these data were not available. It was interesting to note that in 11 cases, it was specified that no anatomical vascular abnormalities were reported in the preoperative workup. In the other articles, this information was not specified. Unfortunately, none of the articles reported the history of previous surgeries of the patients (except for those related to the underlying condition, such as diagnostic laparoscopy before the Interval Debulking Surgery, IDS, or Secondary Cytoreduction Surgery, SCS, before tertiary), despite this being a crucial piece of information for assessing the risk of vascular complications. Of the 40 vascular complications identified, 33 were intraoperative complications, and seven were postoperative. In the group of postoperative complications, in all cases (n = 6) where the complication was described in the article, it was classified as grade 3b according to the Clavien–Dindo classification [22]. In one case, it was not possible to extract the data. The classification grade was assigned retrospectively by the authors of this review based on the description of the complication and its management; this information was not present in any of the articles included.

Regarding the type of surgery, four complications occurred during recurrence: two SCS for lymph node recurrences and one tertiary cytoreductive surgery for a second recurrence in the perisplenic area. The last case concerned a patient with multiple previous relapses, who underwent surgery for the seventh one, in the pelvic area. In the other 19 cases, it was primary ovarian cancer surgery. In particular, six were staging surgeries, three were primary debulking surgeries (PDS), one was an IDS, one was a fertility-sparing staging surgery, three were restaging surgeries, and in four other cases, the data were not known. In one article, including 16 vascular complications, the cases were described as advanced or recurrent ovarian cancer with perihepatic liver involvement without specifying the case-by-case type of surgery (PDS, IDS, and SCS). Among the procedures that caused vascular complications, forty-eight were performed laparoscopically, eight via laparotomy, and none of these surgical operations were initially performed robotically. There were 13 intraoperative laparoscopic complications. Of these, in two cases, the need for laparotomic conversion was not described; in one case, a conversion was necessary, while in ten cases, it was not. As for the specific surgical procedure during which the complication occurred, in six cases it was a lymphadenectomy, in one case it was a rectosigmoid resection, in one case the complication occurred during the mobilization of the perisplenic recurrence, in one case the diagnosis was made post mortem, so it was not possible to determine the exact surgical procedure involved. In 16 cases, the specific surgical procedure was not specified, but it was evident that the procedures involved the perihepatic area. In the other cases (n = 14), the data could not be extracted from the article. In the case report concerning the post mortem diagnosis [8], since the damaged vessel was the right external iliac artery and it was a staging procedure, we can assume that the damage occurred, as in most other cases, during a lymphadenectomy.

We therefore sought to identify another crucial piece of information: which specific vessel was damaged. We found that the injuries involved both the arterial and venous systems. The vessels involved were the external iliac artery (n = 7), the left common iliac artery (n = 1), the abdominal aorta (n = 2), and the inferior mesenteric artery (n = 1) on the arterial side, while on the venous side, the external iliac vein (n = 1), the internal iliac vein (n = 1), the common iliac vein (n = 1), the inferior vena cava (n = 8), the lumbar vein (n = 1), the portal vein (n = 4), the main hepatic veins (n = 5), the intra-hepatic venous branches (n = 2), and the epigastric vein (n = 1) were involved. In one case, the bleeding was caused by the rupture of the splenic capsule. In four cases, the data were not reported.

Regarding the type of injury, these were very heterogeneous: three ruptures of a pseudoaneurysm, one of which was associated with an aorto-ureteral fistula post-end-to-end ureteral anastomosis, and one with a ureteral-arterial fistula, electrocautery injury, thermal injury with bipolar forceps, partial thickness injury involving the muscularis layer (scoop-like), two perforations, a laceration, iliac-colonic fistula, injury of the splenic capsule, and two spontaneous ruptures of the involved vessel. In the other cases, the type of damage was not described. Concerning blood loss resulting from the various reported injuries, although this information is important, the data appear to be neither complete nor consistent. Blood loss was reported accurately in only four instances: 3640 mL, 1200 mL, 1000 mL, and 420 mL. In one case, the description provided was limited to a designation of “massive acute hemorrhage” without accompanying quantitative specification. Regarding the scoop-like injury, which constituted a partial and not a full-thickness injury, there was no significant blood loss attributable to that specific lesion. In the other cases, blood loss was either not reported or the data remained inaccessible or difficult to extrapolate. The management approach was tailored to the individual cases. It encompassed ligation of the vessel alongside femoral artery bypass, the placement of an aortic endograft, clamping utilizing endo clinch in conjunction with 10 mm laparoscopic titanium clips, suturing of the vessel employing 4-0 or 5-0 Prolene, followed by rapid clamping and suturing with two 3-0 Vicryl stitches, placing of a left common iliac artery stent and aneurysm repair. Additionally, the procedures included packing of the pelvis, succeeded by venography and vessel occlusion utilizing a 4 mm Nester^®^ embolization coil, as well as iliac femoral bypass featuring a saphenous graft. An overlay of autogenous tissue patch, specifically a segment of rectus abdominis fascia, was secured in one of the cases with four interrupted 5-0 Polydioxanone sutures. Finally, also a transcatheter arterial embolization was described, where ligations were performed without necessitating a crossover bypass to sustain perfusion alongside. In one instance, the paper addresses immediate management without delineating the measures undertaken. Conversely, for the other cases, the management protocols were not clarified. The involvement of vascular surgeons was noted in two cases, while interventional radiologists participated in other three cases. In three instances, it is stated that no other specialists were engaged, whereas the remaining articles do not clarify the involvement or absence of additional specialists. Data regarding transfusions of red blood cells or other components were also pursued. One patient was administered 2800 mL of red blood cells, 2400 mL of fresh frozen plasma, and 400 mL of platelets; another one received 19 units of blood products. In three cases, transfusions were deemed unnecessary. In the remaining instances, this information is either unspecified or could not be appropriately extrapolated.

## 4. Discussion

This review has several limitations that must be acknowledged. First, most of the included studies are case reports, case series, or monocentric retrospective analyses, which inherently carry a higher risk of bias and limited generalizability. The lack of large-scale, multicenter studies means that the reported data may not accurately reflect the true incidence, characteristics, and management outcomes of vascular complications in ovarian cancer surgery. Additionally, the studies often lack standardization in reporting key variables, such as the type of vascular injury, the management strategies employed, and patient outcomes. This heterogeneity makes it challenging to draw definitive conclusions or establish evidence-based guidelines. A further limitation lies in the potential publication bias, as rare or severe complications are more likely to be reported, while minor or uneventful cases may go unrecorded. The absence of robust preoperative and postoperative data, such as detailed imaging or long-term follow-up, further restricts the depth of analysis. These limitations underscore the need for future multicenter, prospective studies that standardize data collection and focus specifically on vascular complications in ovarian cancer surgery. This review unequivocally demonstrates that research on vascular complications associated with ovarian carcinoma surgery is limited in both quantity and quality. Given the infrequency of these complications, researchers have predominantly chosen to examine them within the broader context of laparoscopic procedures or gynecological surgeries in general rather than concentrating specifically on surgeries related to ovarian carcinoma. Consequently, the inadequacy of data and standardization significantly hampers the establishment of evidence-based guidelines that could support surgeons in the comprehensive management of vascular complications associated with ovarian cancer surgery.

Ovarian cancer surgery is characterized by a comparatively low risk of vascular complications, as it involves an intraperitoneal pathology, with nearly all surgical interventions occurring at the peritoneal–intraperitoneal interface. There are some exceptions: debulking of lymphadenopathy in advanced ovarian cancer and recurrences, often retroperitoneal, usually in patients who have undergone surgery or other treatments, leading to higher surgical complication risks. Additional instances of potential vascular injury in the context of ovarian cancer surgery encompass surgical staging in early ovarian cancer, which includes pelvic and para-aortic lymph node dissection, as well as debulking of the upper abdomen. This may involve the mobilization of the liver, diaphragmatic procedures, cholecystectomy, stomach mobilization, omentectomy, splenectomy, pancreatectomy, nephrectomy, etc.) [37,38,39,40].

The main risk factors identified in the literature for vascular complications include BMI (not only obesity but also excessive thinness due to the closer proximity of the vessels to the abdominal wall and the consequent risk of injury during access), previous surgery, older age, presence of adhesions, vascular anomalies (hence the importance of a correct preoperative imaging assessment), and performing the intervention in low-volume patient centers [7,41,42,43,44,45].

Furthermore, certain traits that may heighten the risk of vascular complications in these surgeries include the tumor’s specific characteristics, which can readily invade major vessels when extending beyond the peritoneum. Additionally, anatomical changes caused by carcinosis or omental cake, previous surgeries, or unrecognized congenital anatomical variations may also contribute to this risk [46]. A study conducted by Klemm et al. in 2004 [47] highlights that vascular abnormalities were identified in 30.2% of patients undergoing laparoscopic infrarenal para-aortic lymphadenectomy for gynecological cancer surgery. Notably, in cadaveric dissections, the percentage was even higher at 44.4%. The observed anomalies included variations in renal, lumbar, and ovarian vessels. If these vascular anomalies are incidental findings, they can potentially increase the risk of complications. These data highlight the importance of having a solid understanding of anatomy and adequately preparing for surgery with optimal imaging. In particular, venous variations are more common than arterial in the pelvis. For example, variations in iliac vein anatomy or unusual venous connections may increase the likelihood of injury during lymphadenectomy or pelvic dissection. Similarly, identifying less common arterial anomalies, such as variations in the obturator artery or additional renal arteries, could help refine surgical approaches [48,49,50].

A comprehensive preoperative evaluation is essential for identifying anatomical anomalies and delineating the extent of tumor involvement with respect to the vascular structures. In this context, Tinelli et al. proposed a classification system for preoperative vascular assessment, establishing a grading scale for disease infiltration that ranges from 1 to 5. Grade 1 pertains to a tumor that approaches the periphery of the major vessels without infiltrating their walls. In contrast, Grade 5 indicates a tumor that entirely encircles and obliterates the major vessels [16].

Moreover, it is well known that previous radiotherapy treatments can lead to fibrotic processes in healthy tissues, which in turn can cause anatomical distortion, obliteration of anatomical planes, and increased difficulty in tissue dissection, as well as issues with wound healing due to an elevated catabolic state. The data concerning chemotherapy are more reassuring but still report delayed wound healing and widespread edema [51,52].

Regrettably, vascular surgery training is not included in the standard curriculum for gynecologic oncology surgeons. Furthermore, a diminishing number of emerging surgeons are obtaining sufficient training in extensive lymphadenectomies due to the growing adoption of sentinel node techniques. Considering that lymphadenectomy is among the procedures with the greatest risk of vascular complications, this significantly affects the abilities of novice surgeons to handle vascular complications. In the future, due to the infrequency of this complications, utilizing 3D visualization systems for immersive virtual reality simulations of vascular problems would be beneficial [53,54,55]. Prospective comparative studies would also prove helpful in examining vascular complications among surgeons with similar experience but varying levels of vascular surgery training (both in live surgery and virtual reality settings) [56,57].

Furthermore, there is no current consensus regarding the management of complications associated with ovarian cancer surgery. The management approach largely depends on the surgeons’ expertise and familiarity with the tools at their disposal. Among the few guidelines addressing vascular complications, the ESGO Peri-operative Management of Advanced Ovarian Cancer Patients Undergoing Debulking Surgery guidelines [14] indicate that a multidisciplinary major hemorrhage protocol should be established in any medical facility conducting ovarian cancer surgeries, with regular assessments and updated reviews. Furthermore, it states that it is advisable to use various local hemostatic agents based on their mechanisms of action, consider abdominal packing as an alternative for managing uncontrollable bleeding, and maintain normothermia and regulate acidosis as essential measures to support hemorrhage management. Blood transfusions and pharmacological agents, such as tranexamic acid, are recommended. Additionally, interventional radiology is considered a viable option for addressing active bleeding or suspected pseudoaneurysms in stable postoperative patients.

**Figure 2:**
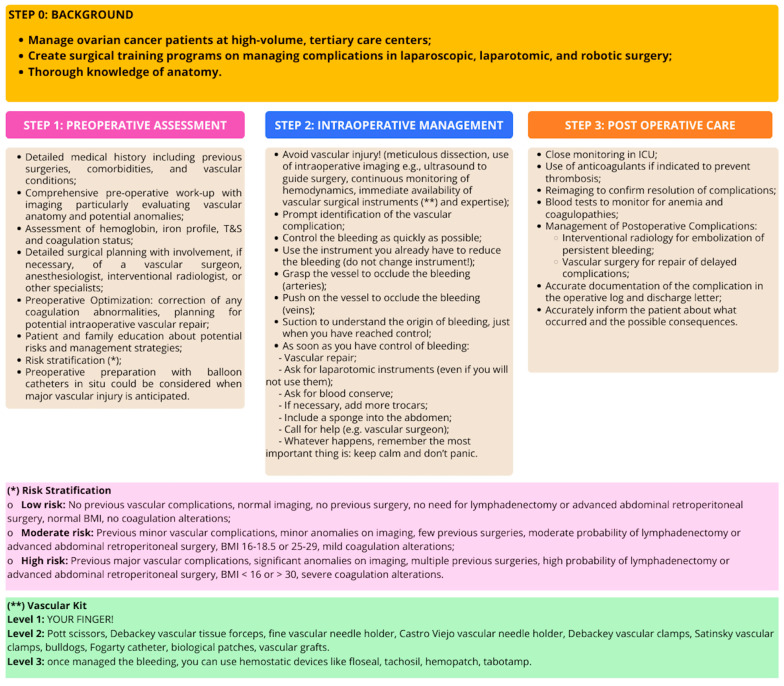
Management algorithm for vascular complications in ovarian cancer surgery.

Consequently, until the literature provides a more evidence-based approach and specific training methods can be routinely implemented, it is imperative that patients with ovarian cancer receive treatment in tertiary centers by surgeons with extensive expertise in both laparoscopic and open surgical techniques. Furthermore, we propose an algorithm designed to assist surgeons in preventing and managing such complications (Figure 2). This algorithm is exclusively derived from expert opinions and the available literature, thereby serving as a basis for developing future evidence-based guidelines. The algorithm consists of the following.

It is important to consider vascular complications proactively, preparing for them preoperatively and postoperatively. A thorough understanding of abdominal anatomy, including variants, is essential. This knowledge helps surgeons know which structures can be sacrificed in emergencies. For example, the entire system of the internal iliac veins can be sacrificed without major complications. Often, the external iliac vein can also be sacrificed, although it can lead to lower limb edema [58]. The vessels that must be preserved are the vena cava above the renal veins, the renal veins, the superior mesenteric vein, and the portal system. On the arterial side, the circumstances vary significantly; nearly all arteries, with few exceptions, must be preserved. For instance, the inferior mesenteric artery is an exception when Riolan’s arc is open. However, if resection becomes necessary, arteries can be reconstructed, highlighting the importance of collaborating with a vascular surgeon in these situations [59]. The first step is the preoperative phase. To be well prepared, it is essential to identify patients who are at greater risk for vascular complications. A crucial step involves obtaining a detailed medical history, which includes prior surgeries, comorbidities, and vascular conditions; conducting a comprehensive preoperative workup with precise imaging to assess vascular anatomy and identify potential anomalies; and evaluating hemoglobin, iron profile, type and screen, and coagulation status. A comprehensive surgical planning process should include, if necessary, the involvement of a vascular surgeon, anesthesiologist, interventional radiologist, or other relevant specialists. Additionally, it is imperative to address any identified issues prior to the surgical procedure, such as blood transfusions or intravenous iron infusions in the event of detected anemia. Preoperative preparation with balloon catheters in situ (inserted by interventional radiologists) could be considered when major vascular injury is foreseen. The use of endografts in the aorta or iliac arteries as a preventive measure represents another strategy to mitigate the risk of massive bleeding during surgery. By placing an endograft, vascular integrity can be preserved, even in cases of inadvertent arterial injury. This approach may be particularly beneficial for high-risk patients where tumor infiltration into the vascular wall is suspected or confirmed. However, the use of preoperative endografts also requires careful consideration of potential complications, such as thrombosis or stent migration, and necessitates close collaboration with vascular surgeons and interventional radiologists. In such cases, it is essential to discuss the case in advance with a multidisciplinary team. Additionally, in some situations, placing a vascular loop around the vessels may be useful, keeping it ready to tie off if needed. Furthermore, the surgical theater and staff must be adequately prepared for any potential intraoperative vascular repairs. Communicating the possible risks and management strategies to the patient and their family is also essential.

Based on the preoperative data, we suggest a risk classification system that requires a more straightforward definition and validation through additional research:

– Low Risk: Patients without prior vascular complications, who present with normal imaging results, have not undergone previous surgical procedures, and do not require lymphadenectomy or advanced abdominal retroperitoneal surgery. These patients should have a normal Body Mass Index (BMI) and exhibit no coagulation irregularities. They should undergo routine preoperative laboratory tests. Basic vascular surgical instruments should be available, but additional resources or multidisciplinary team members may not be immediately necessary. It is not necessary to pre-request blood units.
– Moderate Risk: Patients with a history of minor vascular complications, presenting with minor imaging anomalies and a limited number of previous surgical interventions. There is a moderate likelihood of requiring lymphadenectomy or advanced abdominal retroperitoneal surgery, with a BMI ranging from 16 to 18.5 or 25 to 29, along with mild coagulation alterations. Ensure preoperative optimization of any mild comorbidities (e.g., borderline coagulation issues or minor anemia). Availability of vascular surgical instruments and a vascular surgeon on call is recommended. Discuss potential complications with the patient and their family preoperatively. Request blood units.
– High Risk: Patients who have experienced major vascular complications, display significant imaging anomalies, and have undergone multiple previous surgeries. There is a high probability of necessitating lymphadenectomy or advanced abdominal retroperitoneal surgery, with a BMI below 16 or exceeding 30, alongside severe coagulation abnormalities alterations. Multidisciplinary preoperative meetings could be useful to plan for complex cases. Correct any significant anemia, coagulopathy, or other comorbidities in advance (e.g., using blood transfusions or intravenous iron). A vascular surgeon should be present or immediately available, and advanced vascular surgical instruments should be on hand. Blood products should be readily available, and intraoperative imaging tools (e.g., ultrasound) may be necessary for real-time guidance. Intensive postoperative care, including anticoagulation and close monitoring for delayed vascular complications, is strongly recommended.

This classification serves as a checklist for risk factors. However, it fails to consider the interactions among these factors themselves, providing only limited support for decision-making. It is crucial for future studies to create validated predictive risk models derived from this proposed classification.

The second step involves intraoperative management. The key principle to remember here is that management is unnecessary if a complication does not manifest. Thus, the focus should be on careful and meticulous dissection, utilizing intraoperative imaging (such as ultrasound) to facilitate surgery, continuously monitoring hemodynamics, and ensuring vascular surgical instruments are readily available. If a complication arises despite preventive efforts, promptly identifying it becomes essential. Then, controlling any bleeding must be carried out as swiftly as possible. Additionally, it is important to remember that during open surgery, the surgeon’s finger—or that of the assistant—serves as the initial means to stop any bleeding. A general and effective approach, particularly relevant in laparoscopic surgery, is to minimize bleeding by utilizing the instrument that the surgeon is currently wielding (without changing instruments). Regarding laparoscopy, a particularly useful technique, especially in cases of venous bleeding, is to increase intra-abdominal pressure to help control the bleeding. Once hemorrhage control is established, it is imperative for the surgeon to ascertain the origin of the bleeding, enlisting the help of the assistant to maintain a clear surgical field using either a suction device or gauze. Upon securing hemostasis, the surgeon must proceed with vascular repair commensurate with the nature of the injury sustained. It is essential to request laparotomic instruments (even if not immediately utilized) and blood units in preparation for potential surgical needs. Recognizing the appropriate moments to convert to laparotomy during laparoscopic or robotic procedures is crucial, as it mitigates unnecessary risk while safeguarding against futile laparotomies. When the circumstances require it, additional trocars may be beneficial. If the situation’s complexity escalates, it is pivotal to summon assistance and, irrespective of the unfolding events, to remain composed and focused, avoiding panic. Caring for any complications continues after surgery (step 3), with careful monitoring—sometimes in the intensive care unit if needed. We may use anticoagulants to prevent thrombosis and run blood tests to keep an eye out for anemia and coagulopathies. If necessary, reimaging can help confirm that complications have been resolved. It is important for the surgeon to be aware that vascular complications can also arise after the procedure. Usually, managing these situations involves working with interventional radiology to embolize any ongoing bleeding or with vascular surgery to address delayed complications. Documenting the complications accurately in the operative log and discharge letter is crucial, and once the patient is stabilized, we strive to inform them about what happened and the potential outcomes and consequences. For the complication’s logging, the ESGO report form for ovarian cancer surgery can be used [60].

To implement the algorithm in clinical practice, a phased approach is recommended. Piloting the algorithm in high-volume surgical centers will help assess feasibility and gather feedback, while periodic updates based on emerging evidence will sustain its relevance. National or international registries can provide insights into its real-world application. Incorporating the algorithm into institutional guidelines, creating checklists, and conducting regular case reviews can support its adoption. Potential barriers to its application include the algorithm’s reliance on expert opinion due to limited high-quality studies and challenges in low-resource settings that lack vascular specialists or advanced technologies. Centralized care in tertiary centers may address these issues.

The findings of this review highlight significant gaps in the literature regarding vascular complications in ovarian cancer surgery, underlining the urgent need for more robust and comprehensive research in this area. Future studies should prioritize multicenter, prospective designs to enhance the generalizability of results and reduce bias associated with small, single-center studies. Such research would allow for the collection of standardized data on the incidence, risk factors, and management strategies, providing a more accurate picture of their impact. Additionally, specific areas require further investigation, such as the role of preoperative imaging in identifying vascular anomalies and the impact of anatomical variations on surgical outcomes. Large-scale studies are also needed to evaluate the effectiveness and feasibility of the proposed management algorithm, including its ability to improve patient outcomes and guide gynecologic surgeons in high-risk scenarios. Moreover, as illustrated by the provided schema, this algorithm does not specifically pertain to ovarian cancer; rather, it can be broadly extended to encompass all gynecological surgeries. Extensive studies on vascular complications in ovarian cancer surgery should be published in the future, allowing this framework to be adapted to address the specific pathology. By maintaining a flexible and comprehensive approach, the current algorithm establishes a robust foundation that can be refined and specialized as new evidence emerges. Finally, future research should explore integrating advanced technologies, such as virtual reality simulations, to enhance surgical training for managing vascular complications. This would help address the current gap in vascular surgery training for gynecologic oncology surgeons.

## 5. Conclusions

In summary, effectively managing vascular complications during ovarian cancer surgery is vital for patient outcomes. The proposed algorithm offers a structured method for anticipating, recognizing, and treating these complications. Furthermore, this review emphasizes the necessity for multicentric studies with large sample sizes to assess the incidence, features, risk factors, and management strategies for vascular complications associated with ovarian cancer surgery. Such research could pinpoint critical aspects of these complications and lead to the implementation of targeted prevention strategies. The management algorithm suggested in this study may be adjusted as new literature becomes available. Lastly, this review reiterates the importance of providing care for ovarian cancer patients at high-volume, tertiary care centers with a multidisciplinary approach and developing surgical training programs that focus on addressing complications in laparoscopic, laparotomic, and robotic surgeries. Advanced technology and artificial intelligence could support this initiative through innovative teaching methods and virtual immersive reality experiences.

## Figures and Tables

**Table 1:** Characteristics of the included studies.

Authors	Year of Publication	Type of Article	Ovarian Cancer Patients Number (n)	Complications Number (n)
Goldstein et al. [21]	1975	Monocentric retrospective	1	1
Garrett et al. [23]	2005	Case report	1	1
Anchala et al. [24]	2010	Letter to the editor	1	1
Fotopoulou et al. [25]	2010	Case report	2	2
Vagnoni et al. [26]	2013	Case report	1	1
Gallotta et al. [27]	2014	Multicentric retrospective	300	6
Bae et al. [28]	2015	Monocentric retrospective	14	1
Yano et al. [29]	2017	Case report	1	1
Tingey et al. [8]	2017	Case report	1	1
Atileh et al. [30]	2018	Research letter	1	1
Jung et al. [31]	2019	Monocentric retrospective	59	1
Cosentino et al. [32]	2019	Case report	1	1
Capozzi et al. [33]	2020	Case report	1	1
Tinelli et al. [10]	2022	Case report	1	1
Cianci et al. [34]	2022	Monocentric retrospective	455	3
Rosati et al. [35]	2024	Monocentric retrospective	615	16
Finch et al. [36]	2024	Case report	1	1

## Data Availability

The raw data supporting the conclusions of this article will be made available by the authors on request.

## Abbreviations

The following abbreviations are used in this manuscript:

BMI	Body Mass Index
IDS	Interval Debulking Surgery
SCS	Secondary Cytoreduction Surgery
PDS	Primary Debulking Surgery
ESGO	European Society of Gynaecological Oncology
